# Eculizumab treatment of atypical haemolytic uraemic syndrome: results from the largest prospective clinical trial to date

**DOI:** 10.1186/cc13295

**Published:** 2014-03-17

**Authors:** LE weekers, JM Campistol, M Espinosa, AO Gaber, J Menne, E Minetti, F Provot, E Rondeau, M Ogawa, CL Bedrosian, M hourmant

**Affiliations:** 1 University of Liège Hospital, Liège, Belgium; 2 Servicio de Nefrología, Hospital Clínic, Barcelona, Spain; 3 Hospital Universitario Reina Sofía, Córdoba, Spain; 4; 4 Methodist Hospital, Houston, TX, USA; 5 Hannover Medical School, Hannover, Germany; 6 Careggi University Hospital, Florence, Italy; 7 CHU Lille, France; 8 Hôpital Tenon, Paris, France; 9 Alexion Pharmaceuticals, Inc., Cheshire, CT, USA; 10 Institut d'ImmunoTransplantation Urologie et Nephrologie, Nantes, France

## Introduction

Atypical haemolytic uraemic syndrome (aHUS) is a rare, life-threatening disease in which patients experience uncontrolled complement activation leading to systemic thrombotic microangiopathy (TMA). We report on the effect of eculizumab (Ecu), a terminal complement inhibitor approved for the treatment of aHUS, in the largest prospective clinical trial in aHUS.

## Methods

Adult aHUS patients (≥18 years) with platelets <150 × 10^9^/l and LDH ≥1.5 ULN were recruited into a 26-week single-arm, phase 2 study evaluating Ecu treatment. Patients with STEC-HUS (Shiga toxin and *E. coli) *and severe ADAMTS13 deficiency (activity <5%) were excluded. Identification of complement mutation was not required for enrolment. The primary endpoint was complete TMA response (platelet and LDH normalisation (>150 × 10^9^/l and <ULN, respectively) and <25% increase in serum creatinine from baseline (BL)). Other efficacy evaluations measured platelet counts and eGFR improvement (by MDRD).

## Results

Forty-one patients were enrolled (mean (SD) age 40.3 (15.3) years; 28 (68%) females), 30 (73%) of whom with first presentation of aHUS (median 2 weeks before Ecu). BL mean (SD) platelets and eGFR were 119 (66.1) x10^9^/l and 17.3 (12.1) ml/minute/1.73 m^2^, respectively. Thirty-eight (93%) patients received Ecu for 26 weeks. Table 1 shows improvements in outcomes. Dialysis was discontinued in 20 (83%) among the 24 patients requiring dialysis at inclusion. Ecu was generally well tolerated, with no deaths or unexpected safety concerns. Meningococcal infections occurred in two patients, one of whom continued treatment.

**Table 1:** Outcomes at 26 weeks in adult aHUS patients receiving Ecu

Complete TMA response, *n *(%) patients	30 (73); 95% CI 57 to 86
Platelet count normalisation, *n *(%) patients	40 (98); 95% CI 87 to 10C
Mean (SD) platelet count increase from baseline, ×10^9^/l	135 (114); *P *< 0.0001
Mean (SD) eGFR increase from baseline ml/minute/1.73 m^2^	29.3 (23.6); *P *< 0.0001

## Conclusion

Results from this large prospective clinical trial in adult patients with aHUS confirm prior trials with Ecu to inhibit complement- mediated TMA, improve renal and haematological outcomes and show the benefit of early diagnosis and treatment. There were no unexpected safety concerns. The trial is ongoing.

